# Quality assessment of a rural population-based cancer registry (PBCR) at Ratnagiri, Maharashtra, India for the years 2017–18

**DOI:** 10.3332/ecancer.2024.1672

**Published:** 2024-02-21

**Authors:** Samyukta Shivshankar, Monika Sarade, Sandip Bhojane, Suvarna Kolekar, Suvarna Patil, Atul Budukh

**Affiliations:** 1 Homi Bhabha National Institute, Training School Complex, Anushakti Nagar, Mumbai 400094, India; 2 Department of Medical Records and Cancer Registries, Centre for Cancer Epidemiology, Tata Memorial Centre, Mumbai 410210, India; 3 Bhaktashreshth Kamalakarpant Laxman Walawalkar Hospital, Dervan, Ratnagiri 415606, Maharashtra, India; a https://orcid.org/0009-0005-9521-9578; b https://orcid.org/0000-0001-6723-802X

**Keywords:** data quality, PBCRs, comparability, validity, completeness, timeliness

## Abstract

**Background:**

Cancer registries are valuable resources for cancer control and research. To justify their purpose, their data should be of satisfactory quality by being comparable internationally, complete in their coverage, valid in their values and timely in reporting. This study aimed to assess the quality of the Ratnagiri Population Based Cancer Registry’s data for the years 2017–18 across the four dimensions of data quality.

**Methods:**

Regarding comparability, the registry procedure was reviewed vis-à-vis the rules they follow for cancer registry operation. We have used four methods for validity: re-abstraction and re-coding, diagnostic criteria methods- like the percentage of microscopically verified (MV%) and of death certificate only (DCO%) cases, missing information like proportion of cases of primary site unknown (PSU%) and internal validity. Semi-quantitative methods were employed for assessing completeness. Timeliness for all years of registry functioning was assessed qualitatively.

**Results:**

The overall accuracy rate of the registry was found to be 91.1% (94.7% for demographic and 88% for tumour details). Mortality to incidence ratios were found to be 0.50 for females and 0.59 for males. MV% was found to be 90.8% for males and 91.5% for females. The average number of sources per case was found to be 1.5. DCO% was found to be 2.7%. PSU% was 7.4%.

**Conclusion:**

We have positive results regarding the data’s validity and comparability, but there is scope for improvement concerning completeness. Continuous training of the registry personnel and monitoring of the registry is recommended.

## Background

The alarming increase in the cancer burden in low and middle-income countries (LMICs), as a product of epidemiological transition, has highlighted the need for strong evidence-based cancer control programmes in these countries [1]. Numerous population-based cancer registries (PBCRs) have been established across India to assess the disease burden and evaluate the cancer control activities implemented in the area [2]. The support of a PBCR as a source of incidence data enables the efficient planning, implementation and evaluation of cancer control programmes [3].

The mere presentation of cancer incidence figures is insufficient; rather, they must be reliable, representative, and relevant for effective utilisation. The quality of the statistics presented by PBCRs greatly affects the usability and rationality of their data [4]. The set-up of continuous and systematic quality control mechanisms is crucial for the smooth running of a cancer registry and to ensure an adequate level of confidence in data used for research [5, 6]. However, in today’s scenario, only one in three countries can report high-quality data on cancer incidence, as per the Global Initiative for Cancer Registration [7]. Good-quality cancer incidence data from LMICs in Asia, Africa and South America is scarce, as expressed in several volumes of the Cancer Incidence in Five Continents [8, 9].

India ranks third worldwide in terms of the number of incidence cases reported [10]. Therefore, it is essential that the data used by India for aetiological study designs, policy planning and programme implementation must be as accurate and representative as possible. Only then would the cancer control programmes planned and executed based on such data be successful [11].

In 2009, Tata Memorial Centre (TMC), a premier institute for cancer care and research in India, established a PBCR in the rural district of Ratnagiri in the Konkan division of Maharashtra state, with the support of and in the premises of Bhaktshreshtha Kamalakarpant Laxman Walawalkar (BKLW) Hospital, Chiplun, Maharashtra [12]. Besides its regular functions as a registry, it also provides support to an ongoing oral cancer screening cluster randomised trial in the district.

As an effort to improve the reporting of rural registries, this study aimed to assess the data quality of the Ratnagiri PBCR, in terms of the four dimensions of data quality as defined by the International Agency for Research on Cancer (IARC): *comparability* of the registry’s procedures with other registries and over time, *completeness* of coverage of cancer cases in the population, *validity* of the information reported and *timeliness* regarding procedure completion and data presentation [13, 14].

## Materials and methods

This study was an observational cross-sectional study, carried out from November 2022 to April 2023, that assessed the data quality of the Ratnagiri PBCR located in coastal Maharashtra, India for the years 2017–18.

**Figure 1.**
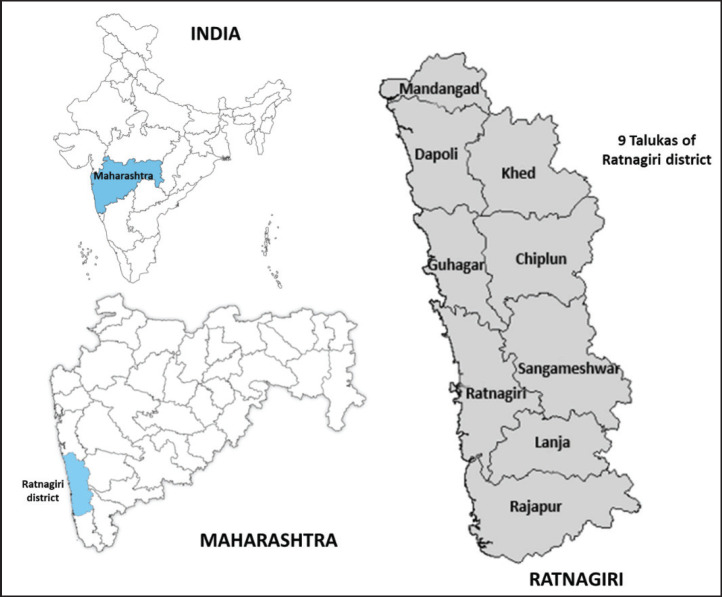
Coverage area of the Ratnagiri PBCR.

Ratnagiri PBCR covers a population of more than 1.6 million, as per the 2011 census of India, which is about 84% rural [15]. The PBCR was established to monitor the outcomes of an ongoing outreach programme back in 2009. Since then, it has been covering all cases arising from Ratnagiri district (which includes 9 talukas (sub-division of a district) and 1,537 villages). The coverage area of the PBCR has been highlighted in Figure 1. The registry employs an active strategy for data collection. Registry personnel visit hospitals, labs and many other sources within and outside the district to obtain information on cancer cases [12, 16]. They also seek information from the death certification office and make village visits for address confirmation and obtaining further details.

The data collected by the registry for the years 2017–18 and past reports were the primary source of data for this study. International standards were referred from various organizational guidelines and their publications including the International Classification of Diseases Tenth Edition (ICD-10), International Classification of Diseases-Oncology Third Edition (ICD-O-3), Cancer Incidence in Five Continents Volume XI (CI5 XI) and IARC technical publication no. 43 [1, 9, 17]. The values of all calculated parameters were compared with those of other Indian registries [18–20].

*Comparability* was assessed by reviewing the definitions used by the registry for incidence cases and incidence date, the system used for classification and coding, and the rules adhered to for handling multiple primaries by the registry. *Completeness* was assessed through the following semi-quantitative methods.

**(i) Historical data methods.** Using already collected data from the registry database, we examined and compared geographic and temporal trends using the following methods:

- Stability of incidence rates over time.
- Comparison of age-adjusted incidence rates of Ratnagiri (2017–18) to those of Barshi (2012–16), both site and sex-wise. Statistically significant differences were flagged. Barshi was chosen as a comparable registry as both registries lie in the same state of Maharashtra and share features of rural demographics [18].
- Incidence of childhood cancers: The age-specific incidence rates of paediatric cancers (0–14 years) were calculated and compared to standards mentioned in CI5 [9].

**(ii) Mortality: incidence ratios (MIRs).** PBCR staff regularly conducts follow up of the registered cancer cases in the region and records date of death accordingly. Additionally, PBCR staff also collect death data from village/municipal death registration office and respective hospital death record departments to check the mortality. Mortality data on cancer by sex and site, for the period from 1st January 2017 to 31st December 2018, were obtained. Then, MIRs for each cancer site, sex-wise, were computed.

**(iii) Average number of sources per case.** The number of cases having one source, two sources and three sources was computed from CanReg5 data, and tabulated to calculate the average number of sources per incident case [21]. The average number of sources per case was calculated on the basis of all 1,897 cases.

The four sets of methods suggested by Bray and Parkin [13] that were used to assess* validity* are as follows.

**(i) Reabstraction and recoding of cases.** Using computer-generated random numbers, a simple random sample of 5% of the cases in the 2017–18 registry database was extracted. Individual reabstraction and recoding were carried out from the Electronic Medical Records of TMC and the registry proforma regarding the ten essential data items defined by MacLennan [22]. Their coding was carried out according to ICD-O-3 [17]. The coding for re-abstracted cases was done by researchers and validated by a senior staff from TMC, Mumbai. Differences between the original abstraction and reabstraction were tabulated and categorised as major and minor disagreements. The overall accuracy rate was calculated for the essential data items.

**(ii) Basis of diagnosis methods.** The proportion of cases that have a morphological/microscopic confirmation by histopathology, cytology, or haematology report (microscopically verified (MV%)) was estimated. The percentage of cases that have no other source of information except for a death certificate pointing to the cause of death or comorbidity as cancer (death certificate only (DCO%)), was estimated.

**(iii) Missing information.** The proportion of cases that have primary site unknown (PSU%) and the proportion of others and unspecified cases (O&U%) were identified from the registry database.

**(iv) Internal consistency check.** The IARC CHECK programme was used to estimate errors within the data [23].

*Timeliness* of all past years of registry functioning was assessed. There are no existing internationally defined standards, but a standard of within 2 years since the close of the registration year was used for comparison, inspired by American registries.

## Results

For the years 2017–18, the Ratnagiri PBCR registered a total of 1,897 incidence cases, out of which 834 cases were males (age-adjusted rate (AAR) 48.8 per 100,000) and 1,063 cases were females (AAR 53.8 per 100,000).

### Comparability

The registry includes only the malignant cases that occur in the district, therefore only behaviour codes 3 and 6 from the ICD-O-3 were considered [17]. Rules for recording incidence dates and multiple primaries were defined by the IARC-IACR recommendations [24]. The classification and coding of tumours’ topographic site, morphology, behaviour, grade and most valid basis of diagnosis were as per the ICD-O-3 [17]. A new code, 8, which is unassigned in the ICD-O-3, was used by the registry to accommodate cases diagnosed by verbal autopsy, a special procedure employed to compensate for the poor availability of cause of death data [25]. Both 0 (the code for DCO) and 8 were used to contribute to DCO cases.

### Completeness

#### By historical data methods

**Figure 2.**
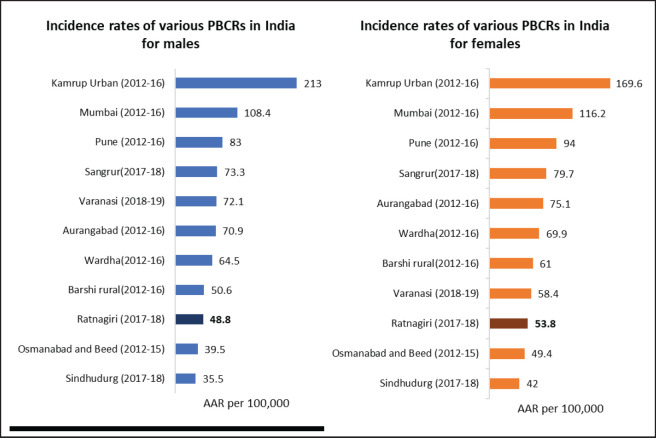
Comparison of AAR per 100,000 of various Indian PBCRs with Ratnagiri PBCR.

*Stability of incidence rates:* The years 2011–14 and 2015–16 showed the lowest reporting in the past 10 years of reporting, as shown in Table 1. Reporting seems to have improved in 2017–18. Figure 2 compares incidence rates of different PBCRs in India with Ratnagiri.

*Rate ratios of incidence rates of leading sites of cancer:* There was no significant difference observed between the overall rates of Ratnagiri and Barshi in both sexes. It is apparent in Table 2 that Ratnagiri reports a significantly higher incidence of mouth cancer than the Barshi registry across both sexes (males; RR 2.45, 95% CI 1.75–3.44 and females; RR = 3.54, 95% CI 2.10–5.95). Also, the rates for cervical cancer were 70% lower for Ratnagiri than Barshi (RR 0.30, 95% CI 0.23–0.40).

**Figure 3.**
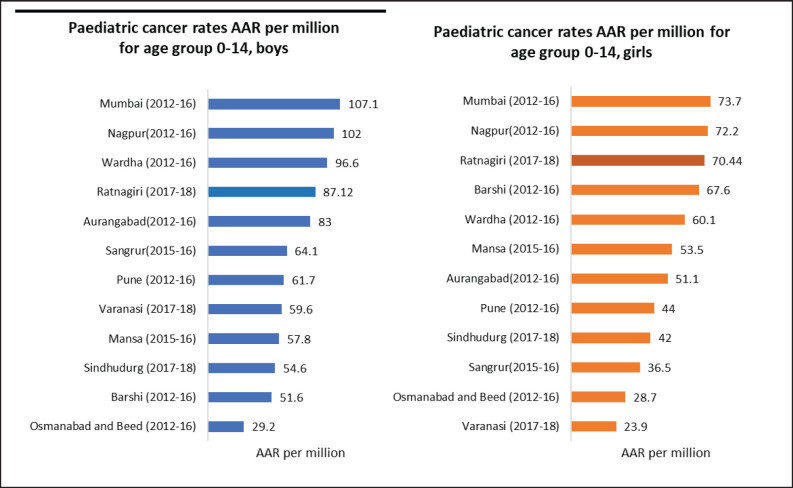
Comparison of paediatric cancer rates in terms of AAR per million for age group 0–14 years across various Indian cancer registries.

*Incidence of childhood cancers:* Except for the age-specific incidence rate for the 0–4 age group in boys, none of the rates for paediatric cancers were within the standard range given in CI5 XI, as can be seen in Table 3. Figure 3 depicts the comparison of incidence rates of paediatric cancers from different PBCRs in India.

#### Mortality to incidence ratios

**Figure 4.**
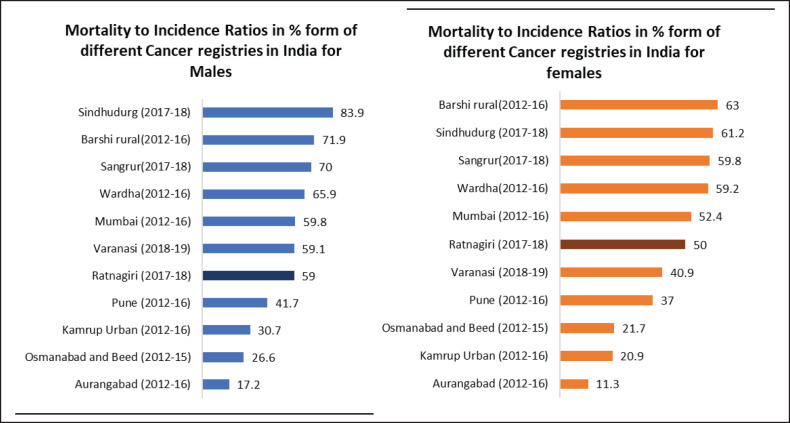
Comparison of MIR across various Indian cancer registries.

The overall MIR for the Ratnagiri PBCR is 59% for males and 50% for females. Site-wise details are shown in Table 4. Figure 4 shows the comparison of MIRs across various Indian registries.

#### No. of sources per case

An average of 1.49 sources have been referred to per case. Out of the 1,897 cases, 1,110 cases were registered using only 1 source.

### Timeliness

The registry has been reporting a delay of almost 4–5 years after the closing of the registration year, as can be seen in Table 1.

### Validity

#### Reabstraction and recoding

A random sample of 101 cases (40 males and 61 females) was re-abstracted for 10 essential variables. Due to the unavailability of certain case records, some variables could not be re-abstracted for all cases. Table 5 mentions the number of cases for which each variable was re-abstracted, the number of disagreements (minor and major), and respective agreement per cent. The highest agreement was observed for name and sex (100%) and the lowest being for topography code (72.3%). The overall accuracy rate was observed to be 91.1%. The accuracy rate for demographic details was found to be 94.7% and for tumour, details were found to be 88%.

#### Basis of diagnosis methods

**Figure 5.**
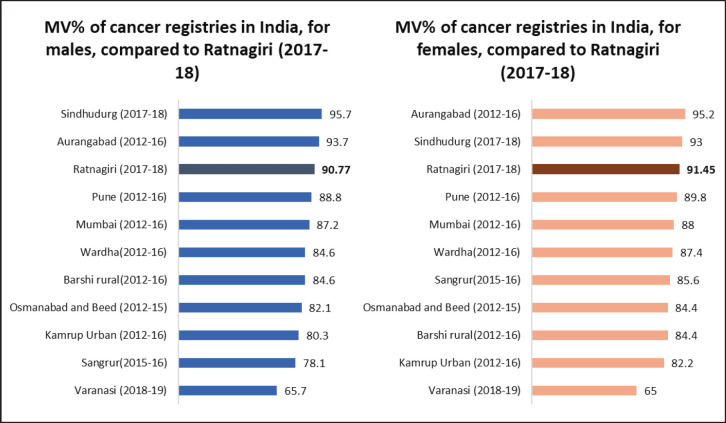
Comparison of MV% across various Indian cancer registries.

In males of Ratnagiri, MV% ranged from 62.5% to 100%, with an overall of 90.8%. In females of Ratnagiri, MV% ranged from 63.6% to 100%, with an overall of 91.5%. For site-wise elucidation, please refer to Table 4. Figure 5 shows the comparison of MV% of various Indian registries.

**Figure 6.**
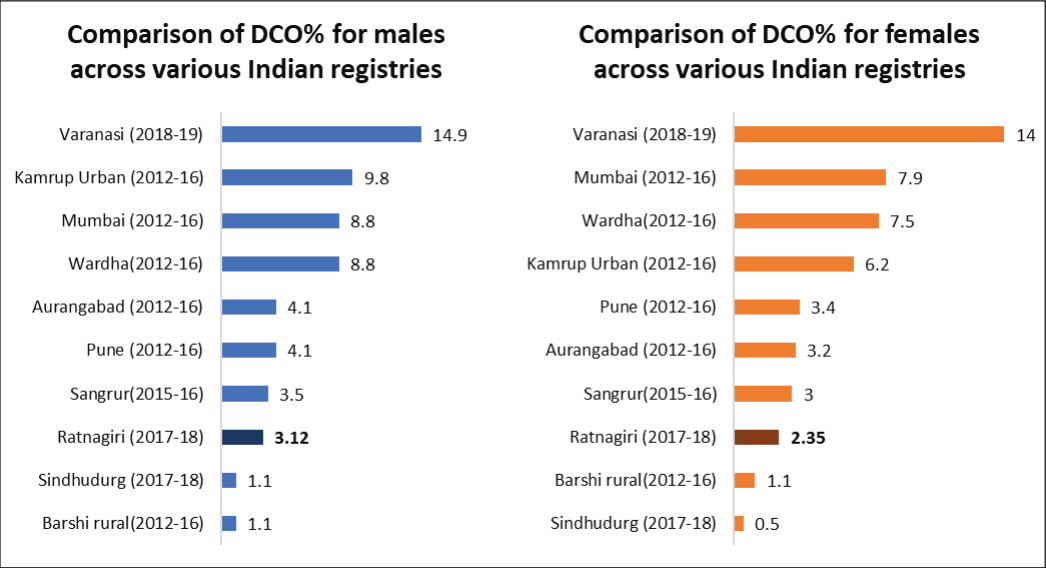
Comparison of DCO% across various Indian cancer registries.

The DCO% estimate of the registry was 2.7% (3.12% for males and 2.35% for females). Figure 6 shows the comparison of DCO% of various Indian registries.

#### Internal validity

IARC CHECK Programme reported 467 errors and 42 warnings. On cross-checking individual records, most of these were found to be due to the internal usage of code 6 and mentions of lymph nodes as primary site, which are added to primary site unknown cases during data presentation.

#### Missing information

There was no missing information within each variable collected indicating internal data validity. Proportion of cases that belonged to C26, C48, C75, C76 and C80, i.e., other and ill-defined sites (O&U%) is 8.3%. Almost half of these cases belonged to geriatric age groups.

The proportion of cases that belonged to C80 i.e., primary site unknown (PSU%), is 7.4%.

## Discussion

**Figure 7.**
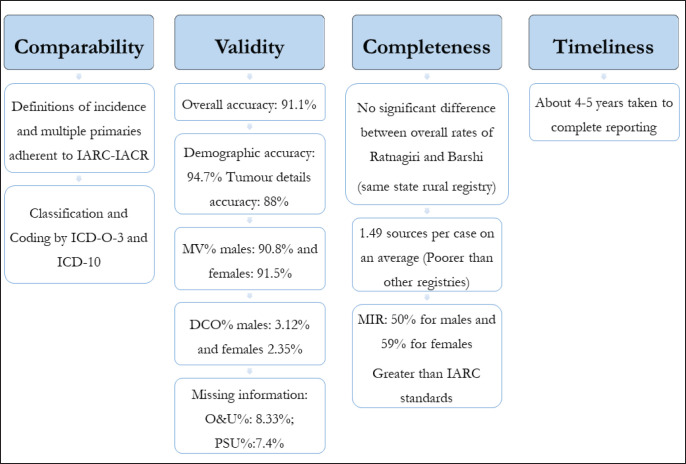
Graphical representation of results of quality control exercise of Ratnagiri PBCR.

Quality assessment of registries on a time-to-time basis ensures able and accurate reporting by them. Only two previous studies from India describe indicators of both completeness and validity of PBCRs: one study assessed the PBCRs covering Chandigarh (Union territory), SAS Nagar, Mansa and Sangrur districts in Punjab, while the other covered Kamrup Urban District in Assam [26, 27]. They too covered rural registries, but their catchment areas were not as populous as Ratnagiri. The overall results of this quality control exercise have been summarised in Figure 7.

Regarding comparability, Ratnagiri PBCR follows all international guidelines with minute modifications to accommodate the rules according to its settings. Thus, the registry was comparable to other registries in terms of definitions of data items like incidence date and classification and coding systems.

Age-adjusted incidence rates of Ratnagiri for the years 2017–18 are lower than most registries of India, as shown in Figure 2. Site-wise AARs are almost three times lesser than the standards mentioned in IARC technical report no. 43, as shown in Table 4. However, the AARs were comparable to another rural PBCR (Barshi) (Table 2). A reason for the low rates observed could be the wide urban-rural difference in cancer incidence in India [28].

Although not within the standard range mentioned in CI5 XI (as shown in Table 3), the registry’s paediatric rates are better than other Indian registries, as seen in Figure 3. Expert consultation provided as a collaboration between TMC and BKLW hospital has increased the accessibility of cancer care in the district and thus, increased the coverage of paediatric cases. Accessibility and availability of medical facilities are prerequisites for a cancer registry so that cancer cases undergo proper diagnosis and treatment at some clinical point of their disease [29].

The registry’s average number of sources per case is low (1.49) as compared to similar rural registries in Punjab like Sangrur (1.7) and Mansa (1.8) [26]. Covering multiple facilities during active case finding can significantly improve the quality of data [5, 30]. The Eastern Cape Cancer Registry, a rural registry in Africa, used 2 years of data collected from four hospitals to conclude that using a well-planned combination of active and passive methods improves case finding by more than 40% [30].

The DCO% estimate of the registry for the year 2017–18, i.e., 2.7% (3.12% for males and 2.35% for females) adheres to both the IARC-IACR standard (DCO less than 20%) and the Indian Council for Medical Research standards [18]. The DCO% estimate is lower than most Indian registries, as shown in Figure 6. Low DCO% indirectly suggests success in case finding [13]. The estimate has also improved as compared to reporting of past years, as shown in Table 1. A reason for this good DCO% estimate could be the employment of verbal autopsy technique in case registration which improved coverage [25].

The overall MV% was higher for females owing to the higher incidence of cancers of internal organs of the female reproductive system. For almost all sites, the MV% estimates were greater than the standards given in IARC technical report no. 43, as shown in Table 4. This is an indication of a biased case-finding procedure that is missing out on sources of radiological and clinical diagnoses [14]. However, as compared to previous years, MV% has decreased (as shown in Table 1), as more sources have been covered this year than in previous years. Nevertheless, such a high MV% indicates good accuracy of tumour details as per IARC standards [13].

The MIR for individual sites being significantly greater than the IARC-IACR India-specific standards (as shown in Table 4) leads to the suspicion that the registry may have missed some incident cases [1]. However, MIR has improved since the initial years of reporting, suggesting that with years, registry coverage has improved. The usage of MIR depends on the accuracy of the death registration system, hence, cannot be used in settings where the death registration is lacking [1]. The usage of verbal autopsy procedures to strengthen our mortality data has enabled us to use this estimate of completeness [25].

The overall accuracy rate was observed to be 91.1% which is lower as compared to other rural registries, like Sangrur (93.4%), and Mansa (94.2%) [26]. Accuracy rate for demographic details was 94.7%, which was lower than that of Mansa (99.3%) and Sangrur (98.6%) [26]. However, the accuracy rate for tumour details was observed to be 88% which is comparable to those observed for the PBCRs of Sangrur (89.1%) and Mansa (89.5%) [26].

Topography coding had the lowest agreement (72.3%) (Table 5). Most disagreements were minor, i.e., a difference was observed in the coding of the subsite [31] due to ignorance of detail. Incidence dates had around 15% disagreements, most of which were minor, i.e., were within the same month [31].

The proportion of missing values was low, which is a good indication as per the standards of IARC-IACR [13]. Almost 50% of cases of primary site unknown belong to the elderly age group, due to poor diagnosis of metastatic disease at advanced age.

Timeliness in registry reporting allows speedy access to cancer information for the benefit of research: affects relevance and reliability [13]. The registry has been consistently late in its reporting, as shown in Table 1. However, there is a paradox in data quality; the faster the data is reported, the more chance it has of being incomplete or having errors [4]. The registry covers a vast geographical area. Often, the registry staff do not receive cooperation from a few sources, hence causing a delay in reporting the data. As cancer is a rare disease, the rates aren’t likely to change significantly in a few years, due to which completeness has been prioritised. However, efforts must made to improve timeliness as well. After this exercise, it has been decided to speed up the process and publish the report in time.

Some objective obstacles impede the adherence of the international standards by the registry, by virtue of its LMIC settings [1]:

- The population is predominantly rural; with low availability and accessibility of medical facilities [15].
- The coverage area of the registry is hilly and houses are placed far apart. It becomes difficult to obtain accurate data regarding personal details and confirm addresses.
- The death registration system isn’t well developed and superstitious people refrain from sharing information about their dead relatives.
- Some hospitals and labs refuse sharing information with the registry personnel as they fear that the privacy of their patients shall be breached.

Keeping in mind all the discrepancies, the registry personnel were informed of the areas of improvement in the registration process. They have been encouraged to periodically assess their data quality internally as well as externally. Building good relationships with the healthcare community is crucial for a cancer registry’s functioning and data quality [5]. Making registry data publicly available on time would inspire the process of quality improvisation [3]. Therefore, the registry must aim to complete its reports within 2 years.

A major limitation of this study was that quantitative methods of estimating completeness couldn’t be used due to the limited availability of survival and mortality data.

On the bright side, this study was the first formal quality assessment of the Ratnagiri PBCR. We attained accurate estimates for various data quality descriptors. We have positive results, in favour of the registry having accurate data, considering DCO%, MV% and O&U%. Through the reabstraction exercise, we have ascertained deficient areas to guide training sessions. We have identified the need for improvisation of the case-finding procedure.

To ensure good data quality, Teppo *et al* [5] emphasized the need for an active research policy and staff proficient in medicine, biostatistics and computer science in a registry. Cancer registries must always strive to maintain a high standard of data quality by being complete and valid at all times, which is, ironically, their greatest challenge as a data collection effort [32].

## Conclusion

The overall quality of the Ratnagiri PBCR’s data is satisfactory and has improved over the years. However, continuous monitoring and periodic quality assessment can further improve the registry’s reporting.

## Conflicts of interest

None declared.

## Funding

None.

## Author contributions

AB designed and supervised the study execution and contributed to writing the manuscript. SS was involved in study execution, literature review, data analysis, and manuscript writing. MS and SB were involved in data collection. MS and SK validated the study results. SP overlooked the study and data collection and lent assistance in writing the manuscript.

## Data disclosure

During this study, tentative data from the year 2022 had been used for analysis. Meanwhile, the 2017-18 PBCR report has been updated later in 2023. Thus, there would be a few differences between the study findings and the registry’s report.

## Ethical declaration

A waiver of consent (reference no. INW/2023/0228) has been obtained from the Institutional Ethics Committee (IEC) of Tata Memorial CentreTMC, Mumbai, India as the study involved no direct involvement of patients.

## Approval

This study has been approved as a dissertation thesis by Homi Bhabha National Institute for partial fulfilment of Ms Samyukta Shivshankar’s MSc degree.

## Figures and Tables

**Table 1.** Comparison of data quality parameters within registry over years of reporting.

Year of registration	Year of Report	Male				Female			
		AAR per 100,000	MV (%)	DCO (%)	MIR (%)	AAR per 100,000	MV (%)	DCO (%)	MIR (%)
2009–10	2014	46.7	95.1	1.2	38.7	46.4	96.2	1	30.2
2011–14	2019	39.2	95.3	0.6	52.6	42.9	95.9	0.9	38.5
2015–16	2020	39.1	94.7	0.6	58.1	39.6	95	0.6	48.5
2017–18	2023	48.8	90.8	3.12	59	53.8	91.5	2.35	50

**Table 2.** AARs, rate ratios and 95% confidence intervals for overall and top five leading sites for both males and females.

Site	AAR per 100,000		Rate ratio	95%CI	
	Ratnagiri (2017–18)	Barshi (2012–16)		Lower limit	Upper limit
Males					
All	48.9	50.6	0.96	0.85	1.09
Mouth	11.5	4.7	2.45^a^	1.75	3.44
Tongue	3.2	2.2	1.43	0.84	2.45
Lung	2.8	1.8	1.54	0.86	2.76
Oesophagus	2.5	3.7	0.67	0.42	1.08
Stomach	2.5	2.3	1.07	0.61	1.88
Females					
All	53.8	61.0	0.88	0.79	0.99
Breast	13.4	12.3	1.09	0.86	1.38
Mouth	5.5	1.6	3.54^ a^	2.10	5.95
Cervix	4.6	15.3	0.30^ a^	0.23	0.40
Ovary	3.9	3.5	1.12	0.72	1.73
Oesophagus	2.5	2.7	0.92	0.56	1.50

a Significant at 5% level

**Table 3.** Childhood cancer incidence in Ratnagiri for the years 2017–18 for both boys and girls.

Age group	Boys			Girls		
	Incidence cases	Age-specific incidence rate per 10^5^	Standard range as per CI5 XI in 10^5^	Incidence cases	Age-specific incidence rate per 10^5^	Standard range as per CI5 XI in 10^5^
0–4	11	15.1^a^	12.6–26.4	7	10.6	12.1–23.7
5–9	5	5.0	8.9–17.9	4	4.3	7.0–13.0
10–14	6	4.3	9.0–17.2	7	5.3	8.2–16.0

a Adheres to CI5 XI standards

**Table 4.** Comparison of MIR, MV% and incidence AAR with India specific standard as given in IARC technical report no. 43.

ICD-10	Cancer site	MV%		MIR in %		AAR per 10^5^	
		Study	Standard	Study	Standard	Study	Standard
Males							
C00–14	Oral cavity and Pharynx	93.4	87.3	53.0	32.0	18.0	19.9
C15	Oesophagus	77.3	76	75.0	48.8	2.5	7.2
C16	Stomach	90.9	72.1	85.0	47.8	2.5	5.6
C18–21	Large bowel	95.5	81.1	79.6	31.9	2.5	5.3
C22	Liver	76.9	76.0	93.0	53.7	0.7	2.9
C25	Pancreas	68.8	59.9	35.0	51.3	0.9	1.7
C32	Larynx	100	81.0	35.0	38.6	1	6
C33–34	Trachea, bronchus and Lung	86	71.3	71.0	49.5	2.8	10.8
C43	Melanoma of skin	100	99.5	62.0	14.6	0.2	0.3
C50	Breast	100	82.8	0.0	31.9	0.2	0.6
C61	Prostate	87.9	78.7	60.0	38.1	1.7	5.1
C62	Testis	88.9	88.2	27.0	17.1	0.6	0.7
C64–66	Kidney, renal pelvis and Ureter	100	91.1	81.0	26.3	0.5	1.6
C67	Bladder	90	78.6	69.0	30.6	1.2	3.3
C70–72	Brain, CNS	90.5	87.7	44.0	32.1	1.1	3.3
C73	Thyroid	100	83.0	36.0	24.0	0.2	1
C81–88, C90	Lymphomas	100	98.1	43.8	34.2	4.1	6
C91–95	Leukaemia	100	93.3	41.0	48.7	2.4	4.1
C00–96 (excluding C44)	All sites excluding (non-melanoma skin cancer)	90.8	80.3	59.0	40.2	48.8	101.6
Females							
C00–14	Oral cavity and Pharynx	96.2	86.6	47.6	27.9	8.9	8.5
C15	Oesophagus	83.3	77.4	60.1	45.8	2.5	4.3
C16	Stomach	80	68.9	80.1	48.7	1	2.7
C18–21	Large bowel	91.7	79.4	37.6	34.6	2.2	4.1
C22	Liver	80	69.8	78.0	56.9	0.5	1.1
C25	Pancreas	63.6	52.1	67.0	49.7	0.5	1
C32	Larynx	100	76.0	83.0	43.1	0.2	0.7
C33–34	Trachea, bronchus and Lung	96.4	71.4	78.0	52.0	1.3	2.5
C43	Melanoma of skin	100	99.4	19.0	13	0.3	0.2
C50	Breast	91.3	85.7	42.0	24.5	13.4	24.1
C53	Cervix uteri	94.7	87.5	44.0	24.9	4.6	17.3
C54–55	Corpus uteri, uterus unspecified	96.9	86.4	39.0	31.6	1.6	2.8
C56	Ovary	88.2	79.0	58.0	32.8	3.9	6
C64–66	Kidney, renal pelvis and Ureter	80.0	89.3	15.0	31.1	0.4	0.7
C67	Bladder	100.0	76.7	55.0	37.0	0.1	0.8
C70–72	Brain, CNS	90.0	86.3	57.0	33.2	0.7	2.1
C73	Thyroid	94.1	84.8	14.0	14.6	0.9	2.4
C81–88, C90	Lymphomas	100	97.8	19.9	35.9	3.0	3.7
C91–95	Leukaemia	100	92.5	38.0	48.2	1.8	2.9
C00–96 (excluding C44)	All sites excluding non-melanoma skin cancer	91.45	82.0	50.3	32.8	53.8	100.3

**Table 5.** Number of reabstractions carried out, number of disagreements and agreement in % for each data variable.

Data variable	No. re-abstracted	Disagreements		Total disagreements	Total agreements	Agreement (%)
		Minor	Major			
Demographic details						
Name	99	0	0	0	99	100.0
Sex	101	0	0	0	101	100.0
Date of birth	101	0	15	15	86	85.2
Taluka code	97	0	6	6	91	93.8
Accuracy (%) for demographic details: 94.7%						
Tumour details						
Incidence date	68	6	4	10	58	85.3
Basis of diagnosis	97	0	8	8	89	91.8
Topography code	101	17	11	28	73	72.3
Morphology code	101	2	6	8	93	92.1
Behaviour	90	0	1	1	89	98.9
Grade	100	0	17	17	83	83.0
Accuracy (%) for tumour details: 88%						
Overall accuracy rate: 91.1%						
